# DAILY EVENING ELECTRONIC MEDIA USE, SEDENTARY BEHAVIORS, AND SLEEP IN LATER LIFE

**DOI:** 10.1093/geroni/igac059.1027

**Published:** 2022-12-20

**Authors:** Yijung Kim, Nicole Richards, Karen Fingerman

**Affiliations:** The University of Texas at Austin, Austin, Texas, United States; The University of Texas, Austin, Austin, Texas, United States; The University of Texas at Austin, Austin, Texas, United States

## Abstract

Sleep complaints and disorders are two of the most common disturbances to health and well-being in later life. This study examined how evening electronic media use and daytime sedentary behaviors affect subsequent sleep hours and perceived sleep quality, and whether consistent sleep hours (i.e., sleep regularity) moderate these associations. Data were drawn from 241 older adults (Mage = 74.02) from the Daily Experiences and Well-being Study who completed ecological momentary assessments and wore an accelerometer for four days on average. A series of conditional fixed-effects models indicated that older adults reported more sleep disturbances on nights following the evening computer use. Sedentary behaviors and evening television viewing were not associated with sleep quantity and quality. Older adults with more consistent hours of bedtime reported better sleep quality regardless of their evening electronic media use and daytime sedentary behaviors, thereby highlighting the importance of sleep regularity in later life.

